# Outsourcing the Remote Management of Cardiac Implantable Electronic Devices: Medical Care Quality Improvement Project

**DOI:** 10.2196/cardio.9815

**Published:** 2019-12-18

**Authors:** Gabriele Giannola, Riccardo Torcivia, Riccardo Airò Farulla, Tommaso Cipolla

**Affiliations:** 1 Ospedale San Raffaele Giglio Cefalù Italy

**Keywords:** remote monitoring, telemonitoring, cardiac implantable electronic devices, implantable defibrillators, pacemaker, implantable cardioverter defibrillator, triage outsourcing, follow-up

## Abstract

**Background:**

Remote management is partially replacing routine follow-up in patients implanted with cardiac implantable electronic devices (CIEDs). Although it reduces clinical staff time compared with standard in-office follow-up, a new definition of roles and responsibilities may be needed to review remote transmissions in an effective, efficient, and timely manner. Whether remote triage may be outsourced to an external remote monitoring center (ERMC) is still unclear.

**Objective:**

The aim of this health care quality improvement project was to evaluate the feasibility of outsourcing remote triage to an ERMC to improve patient care and health care resource utilization.

**Methods:**

Patients (N=153) with implanted CIEDs were followed up for 8 months. An ERMC composed of nurses and physicians reviewed remote transmissions daily following a specific remote monitoring (RM) protocol. A 6-month benchmarking phase where patients’ transmissions were managed directly by hospital staff was evaluated as a term of comparison.

**Results:**

A total of 654 transmissions were recorded in the RM system and managed by the ERMC team within 2 working days, showing a significant time reduction compared with standard RM management (100% vs 11%, respectively, within 2 days; *P*<.001). A total of 84.3% (551/654) of the transmissions did not include a prioritized event and did not require escalation to the hospital clinician. High priority was assigned to 2.3% (15/654) of transmissions, which were communicated to the hospital team by email within 1 working day. Nonurgent device status events occurred in 88 cases and were communicated to the hospital within 2 working days. Of these, 11% (10/88) were followed by a hospitalization.

**Conclusions:**

The outsourcing of RM management to an ERMC safely provides efficacy and efficiency gains in patients’ care compared with a standard in-hospital management. Moreover, the externalization of RM management could be a key tool for saving dedicated staff and facility time with possible positive economic impact.

**Trial Registration:**

ClinicalTrials.gov NCT01007474; http://clinicaltrials.gov/ct2/show/NCT01007474

## Introduction

Remote monitoring (RM) management of patients implanted with cardiac implantable electronic devices (CIEDs) (such as implantable defibrillators) enables early detection of clinically relevant events and complications while partially replacing routine follow-up [1-5]. A number of studies have recently demonstrated that RM reduces the total number of in-office visits [4,6], without negative effects on patient outcome [7,8]; some studies have also shown the positive clinical impact of RM [9-12]. Although RM can reduce clinical staff time compared with standard in-office follow-up [13-15], organizational workflow changes and a new definition of roles and responsibilities may be needed to review remote transmissions in an effective, efficient, and timely manner [16]. A model where nurses might have the responsibility for screening the transmission reports and for discriminating the ones that possibly require clinical escalation and where physicians interpret and document the most critical remote reports, may, in fact, rationalize and optimize the in-clinic daily practice, in the sense of a more extensive and differentiated role organization. Considering that most RM data do not require any clinical escalation [1,14], it has been hypothesized that the remote triage may be outsourced to an external remote monitoring center (ERMC) composed of nurses and physicians skilled to interpret CIED data and to troubleshoot CIED-related problems, resulting in the optimization of the time allocation of highly skilled health care professionals [17].

Efficient allocation of health care professionals’ time is crucial due to the limited resources available for RM activities today and prospectively in the future, given the patient population growth and accompanying follow-up burden [18]. Outsourcing part of the RM activities could, therefore, have a positive impact on both the health care system and patient care [19].

The aim of this quality improvement project is to evaluate the feasibility of outsourcing the triage of CIED remote follow-up in the management of relevant clinical and technical events in a timely manner. We would like to assess if this approach is safe, effective, and efficient and to evaluate the implications in hospital resource utilization. Our purpose, in other words, is to demonstrate that the proposed triage model consents prompt event management, completeness of remote transmission review, and ability in detecting and prioritizing events (efficacy and safety) and that it might imply a reduction in the use of hospital resources required for daily remote CIED management (efficiency).

## Methods

### Project Design and Patient Population

From April 2016 to December 2016, an ERMC composed of 1 trained nurse and 1 supporting physician (HTN Spa, Brescia, Italy) performed daily reviews of remote transmissions from 153 CIED patients implanted in the hospital, S Raffaele Giglio Hospital of Cefalù (Italy): 62 single- or dual-chamber pacemakers (IPGs), 15 single- or dual-chamber implantable cardioverter defibrillators (ICDs), and 76 cardiac resynchronization therapy defibrillators (CRT-Ds). The presented experience is included in the validation effort of Medtronic FOCUSON, a service aiming to save time for health care professionals to enable a higher quality of care. The FOCUSON service is built around a highly skilled team that classifies transmitted patient data based on agreed protocol and promptly notifies the physician, allowing efficient and effective patient treatment.

All consecutive patients enrolled in the CareLink network (CLN) in the considered time frame were considered for this analysis. CLN is an internet-based service that provides device-related and physiologic patient data similar to data that formerly required an office visit, together with training and support services. The key component of the CLN is the CareLink monitor, an in-home monitor for patients who have received a Medtronic implanted cardiac device. All patients were included in the ClinicalService project (ClinicalTrials.gov, NCT01007474). This medical care quality improvement project was approved by the medical director and conforms to the principles outlined in the Declaration of Helsinki. Each patient provided informed consent for data collection and analysis. The activity is based on a well-defined legal framework, where the parts agree on responsibility, safety requirements, data ownership, data managing, and compliance and compare current remote management models.

As standard practice before outsourcing, there were 3 physicians performing electrophysiology and ambulatory activities. No nurse was dedicated to the ambulatory service, so RM relied on physicians only. All staff were well trained to manage RM activities, even in the absence of a prespecified shared protocol. Despite RM being considered as an important part of clinical practice, remote follow-up was often carried out in the middle of other activities in free time slots. Patients usually transmitted data 3 times per year, with a specific date scheduled by the physician during the annual in-office visit. These routine, scheduled, remote device interrogations were structured to mirror in-office device checks. Prespecified alerts related to device functionality and clinical events (called CareAlerts) were activated and were able to trigger automatic transmissions, for the purpose of emergency clinical and technical RM of patients implanted with a device with wireless capabilities, but without any check planned for lost transmissions or disconnected monitors.

### The New Remote Management Model

#### External Remote Monitoring Center Staff Management

**Figure 1:**
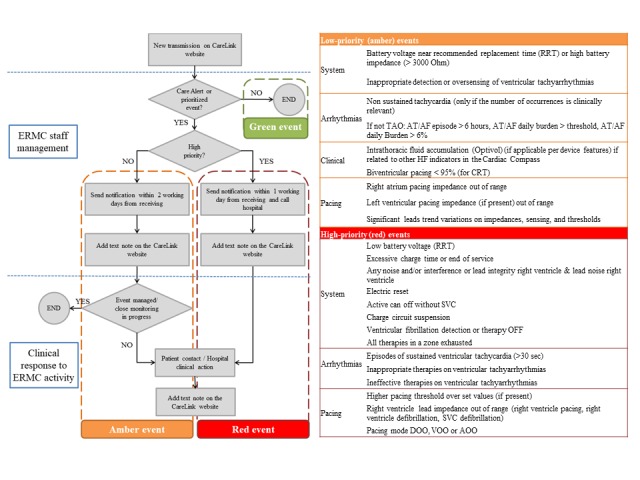
Remote management flowchart. Green events are all transmissions not reporting device detections listed as low or high priority. In case of missed scheduled transmissions or disconnected monitors, the external remote monitoring center (ERMC) inform the technical team responsible for contacting the patient. RRT: recommended replacement time; TAO: oral anticoagulation therapy; AT/AF: atrial tachyarrhythmia/atrial fibrillation; CRT: cardiac resynchronization therapy; SVC: superior vena cava. DOO, VOO, and AOO are programming modes.

The patients included in the service were enrolled by the health care provider in CLN, and details from the patient file as well as their identification numbers were recorded. This anonymous patient identification number was used in all formal communications between ERMC and the health care providers. Patients’ clinical history (eg, implant indication, cardiomyopathy etiology, and atrial fibrillation history) and relevant information (eg, pacemaker dependency, drug therapy with a special attention to oral anticoagulation therapy, and implanted device and leads details) were available for the monitoring center through the *Comments and Notes* field of the CareLink website. Periodic transmissions were scheduled every 3 months or per individual patient needs (eg, to monitor the evolution of a clinical event or to evaluate the battery status in the presence of battery voltage near recommended replacement time). A shared protocol of transmissions review and reporting was defined in agreement with our hospital staff and the ERMC nurse, and the supporting physicians were accurately trained on its application. A daily check to the CareLink website was mandatory (with exclusion of weekends and bank holidays). The protocol required that all transmissions had to be reviewed by ERMC within 1 working day from when they appear on the CareLink website. A flowchart describing timings, roles, and responsibilities was agreed between the hospital physicians and the ERMC staff (Figure 1).

With the aim of effectively reviewing transmissions and managing all possible clinically and technically relevant events, a transmission color-code classification was predefined by the hospital physicians to prioritize device clinical and technical conditions. The different types of relevant events related to each color code are represented in Figure 1. Depending on the priority level assigned to the transmission, the flowchart indicated the maximum time to report the permitted and required modality of communication to the hospital. In particular, in case of high-priority events (red transmissions), the hospital was to be informed by email and phone within 1 working day, whereas for low-priority events (amber transmissions), the protocol planned an email communication within 2 working days. No action was required when the transmission did not contain any prioritized event (green transmissions). When transmissions contained data fulfilling more than 1 color code, the transmissions were managed by using the highest priority color code. In case of an actionable transmission (red or amber), a note was added to the related transmission on the CareLink website. Using this method, both the ERMC nurses and physicians and the hospital staff had the same Web-based clinical repository available at patient level. In case of missed scheduled transmissions or disconnected monitors, the ERMC was instructed to inform the technical team responsible for contacting the patient.

### Clinical Response to External Remote Monitoring Center Activity

On the basis of the received RM data, the clinical response was at the discretion of the involved clinicians. When the patient had to be contacted, a standardized telephone interview was conducted by the medical staff to evaluate the patient’s health condition (worsened dyspnea, increased weight, patient’s compliance with the medical therapy, etc). In addition to the interview details, the hospital staff reported all follow-up clinical actions on the CareLink website. In some cases, prioritized events would not require any action, for example, in case of an event already managed with the appropriate therapy (eg, atrial arrhythmias with optimized drug therapy, intrathoracic fluid accumulation, and other events previously known to the staff), or for which clinicians would rather wait to monitor the status of the event.

### Research Objectives and Outcome Measures, Efficacy, and Safety

The aim of this health care quality improvement project was to assess if outsourcing the triage of CIEDs’ remote follow-up is safe, effective, and efficient to manage relevant clinical and technical events in a timely manner and improve hospital resource utilization. Time to review all transmissions and time to report prioritized events, according to the protocol flowchart, were considered as end points for efficacy and safety.

### Efficiency: Comparison With the Benchmark Phase

Efficiency was defined as the ability to improve transmission review and event analysis with reduced hospital resources and was evaluated, in the same recipient of patients, through the comparison with the standard practice of the same hospital in the 6 months preceding the project.

### Statistical Analysis

Continuous data were summarized as mean and SD or median and the first and the third quartiles (Q1-Q3), categorical data as counts and percentages. Differences in proportions were compared by applying chi-square analysis. Continuous Gaussian variables were compared by the Student *t* test for independent samples, whereas skewed distributions were compared using the Mann-Whitney nonparametric test. To represent the time distributions, box-and-whiskers plots were used. The transmission rates and their 95% CIs were reported. For the scope of the timing analysis, 45 out of 654 (6.9%) transmissions were excluded, as they occurred outside the review time defined in the protocol. The comparison of the number of transmissions reviewed by ERMC in the monitoring center phase with respect to the benchmark phase was performed by means of a Poisson model. Comparison was performed on the subset of patients included in both the monitoring center and standard practice phases (no differences between those patients and the full population were found). The incidence rate ratio (IRR) was reported together with its 95% CI. Missing data were not inputted into any of the analysis. The rate of transmissions, the detected event, as well as time to review all transmissions and time to report prioritized events were retrospectively retrieved from the CLN database for both the ERMC and benchmark phases. All results will be reported for the whole population and separately by device type, as RM protocol may vary according to patients’ treatment indication and to the implanted device.

An alpha level of .05 was considered for each test. All statistical analyses were performed using SAS 9.4 version software (SAS Institute Inc, Cary, NC, USA).

## Results

### Patients

A total of 153 patients with an implanted CIED were included in the project and followed remotely on the CareLink RM network (Medtronic, Minneapolis, MN) for 8.4 (SD 1.1) months, with a total follow-up period of 107 years.

Demographics and baseline patient characteristics are presented in Table 1. Mean age of inclusion was 68 (SD 11) years, with 73.2% (112/153) male patients. Considering device type, 49.7% (76/153) of patients had a CRT-D implanted, whereas 9.8% (15/153) were implanted with single- or dual-chamber ICDs and 40.5% (62/153) with an IPG (of which only 1 was a CRT-P [cardiac resynchronization therapy pacemaker]).

### Efficacy and Safety

#### Transmission Management

From April 2016 to December 2016, 654 transmissions were recorded and reviewed by ERMC corresponding to 613 (95% CI 568-662) transmissions for 100 patient-years. In particular, CRT-D devices transmitted more than the other CIEDs, with 802 (95% CI 729-882) transmissions per 100 patient-years. Transmissions with prioritized events represented 15.7% of the total transmissions, with 82.5 (95% CI: 66.9-102) amber transmissions and 14.1 (95% CI 8.5-23.3) red transmissions per 100 patient-years (Table 2).

**Table 1:** Demographics and baseline patient characteristics.

Patient characteristics		Total (N=153)	CRT-D^a^ (N=76)	ICD^b^ (N=15)	IPG+CRT-P^c^ (N=62)
**Demographics**					
	Age at first implant (years), mean (SD)	68 (11)	69 (9)	64 (13)	68 (13)
	Male, n (%)	112 (73.2)	55 (72)	15 (100)	42 (68)
**Medical history** **, n (%)**					
	Ischemic cardiopathy	56 (37)	31 (41)	11 (73)	14 (23)
	Acute myocardial infarction	35 (23)	28 (37)	7 (47)	0 (0 )
	History of heart failure	107 (70.0)	67 (88)	5 (27)	35 (56)
	New York Heart Association III-IV	54 (35)	56 (74)	2 (13)	0 (0)
	History of ventricular tachycardia/ventricular fibrillation	36 (24)	27 (36)	5 (33)	4 (7)
	Ventricular fibrillation/flutter	2 (1)	2 (3)	0 (0)	0 (0)
	History of atrial tachycardia/atrial fibrillation	72 (47)	18 (24)	3 (20)	51 (82)
	Left bundle branch block	54 (35)	46 (61)	0 (0)	8 (13)
	History of stroke/transischemic attack	10 (7)	6 (8)	4 (27)	0 (0)
	Diabetes	41 (27)	19 (25)	4 (27)	18 (29)
**Medications at baseline^d^, n (%)**					
	Beta-blocker	75 (61)	54 (75)	7 (64)	14 (34)
	Diuretic	74 (60)	56 (78)	8 (73)	10 (24)
	Antiplatelet	18 (15)	15 (21)	0 (0)	3 (7)
	Oral anticoagulants	21 (17)	16 (22)	2 (18)	3 (7)
	Amiodaron	4 (3)	4 (6)	0 (0)	0 (0)
	Calcio-antagonist	6 (5)	3 (4)	0 (0)	3 (7)
	Angiotensin-converting enzyme-inhibitor/angiotensin receptor blockers 2	51 (41)	39 (54)	5 (46)	7 (17)
	Digitalis	1 (1)	1 (1)	0 (0)	0 (0)
**Implantation time^e^, n (%)**					
	Less than 12 months	29 (20)	26 (36)	2 (13)	1 (2)
	12-36 months	59 (41)	43 (59)	7 (47)	9 (16)
	More than 36 months	57 (39)	4 (6)	6 (40)	47 (83)

^a^CRT-D: cardiac resynchronization therapy defibrillator.

^b^ICD: single- or dual-chamber implantable cardioverter defibrillator.

^c^IPG + CRT-P: single- or dual-chamber pacemaker + cardiac resynchronization therapy pacemaker.

^d^124 patients with data about medication at baseline, 72 CTR-Ds, 11 ICDs, and 41 IPGs.

^e^145 patients with available date of implant, 73 CRT-Ds, 15 ICDs, and 57 IPGs.

**Table 2:** Rate of transmission, overall and by priority.

Transmission priority		All (n=153, 107 patient-years)	CRT-D^a^ (n=76, 53 patient-years)	ICD^b^ (n=15, 10 patient-years)	IPG^c^ (n=61, 61 patient-years)	CRT-P^d^ (n=1, 1 patient-year)
**All transmission**						
	Transmissions, n	654	426	50	176	2
	Annual rate of transmissions per 100 patient-years (95% CI)	613 (568-662)	802 (729-882)	504 (382-665)	410 (354-476)	—^e^
**No prioritized event (green transmissions)**						
	Transmissions, n (%)	551 (84.3)	364 (85.4)	40 (80.0)	147 (83.5)	0 (0.0)
	Patients with green transmission, n	141	67	14	60	0
	Annual rate of transmissions per 100 patient-years (95% CI)	517 (475-561)	648 (585-718)	393 (288-536)	333 (283-391)	—
**Low-priority events (amber transmissions)**						
	Transmissions, n (%)	88 (13.5)	53 (12.4)	9 (18.0)	24 (13.6)	2 (100)
	Patients with amber transmission, n	50	28	4	17	1
	Annual rate of transmissions per 100 patient-years (95% CI)	82.5 (66.9-102)	94.4 (72.1-124)	88.4 (46.0-170)	54.3 (36.4-81.1)	277 (69.2-1106)
**High priority events (red transmissions)**						
	Transmissions, n (%)	15 (2.2)	9 (2.2)	1 (2.0)	5 (1.7)	0 (0.0)
	Patients with red transmission, n	9	5	1	3	0
	Annual rate of transmissions per 100 patient-years (95% CI)	14.1 (8.5-23.3)	16.0 (8.3-30.8)	9.8 (1.4-69.7)	11.3 (4.7-27.2)	—

^a^CRT-D: cardiac resynchronization therapy defibrillator.

^b^ICD: single- or dual-chamber implantable cardioverter defibrillator.

^c^IPG: single- or dual-chamber pacemaker.

^d^CRT-P: cardiac resynchronization therapy pacemaker.

^e^Not applicable.

**Figure 2:**
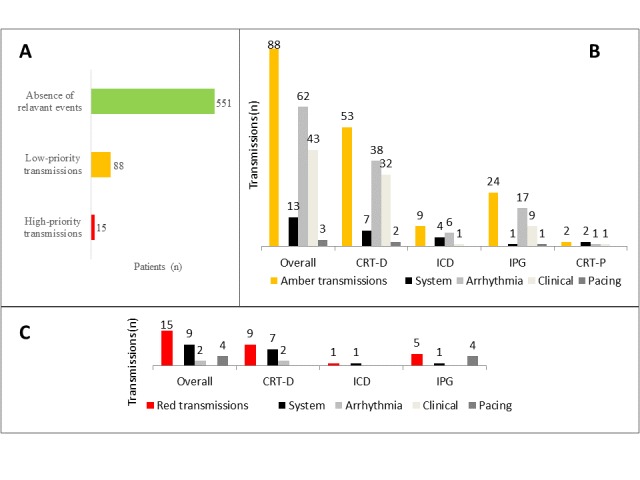
(A) Distribution of transmission by priority; (B) low-priority detected events; and (C) high-priority detected events. CRT-D: cardiac resynchronization therapy defibrillator, ICD: single- or dual-chamber implantable cardioverter defibrillator, IPG: single- or dual-chamber pacemaker, CRT-P: cardiac resynchronization therapy pacemaker.

Most of the amber transmissions reported arrhythmia events, whereas two-thirds of the red transmissions presented system issues (Figure 2). Almost all transmissions (99.7%) were reviewed within 1 working day and 86.7% within 24 hours, considering some transmissions occurred outside working hours, as defined in the protocol. Our analysis did not show any predictors of low- or high-priority transmissions, neither considering implanted device type (when compared with the others, IRR for CRT-D was 1.52 [95% CI 0.93-2.50; *P*=.095] and IRR for IPG+CRT-P was 0.63 [95% CI 0.37-1.05; *P*=.078]) nor considering other risk factors (all *P*>.1).

#### Prioritized Events Communication

**Figure 3:**
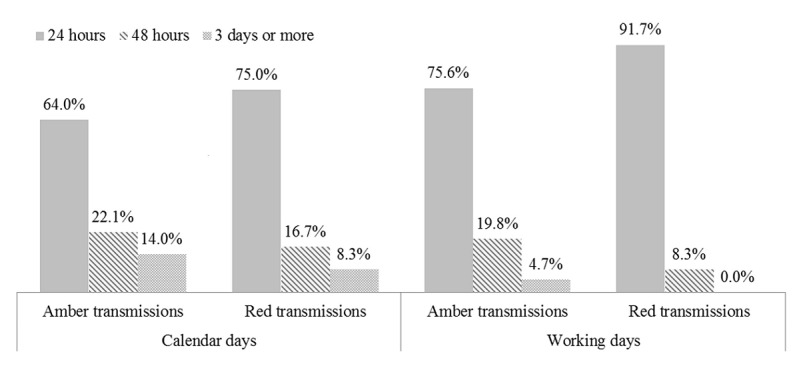
Time from transmission to communication with the hospital.

Following the protocol, ERMC communicated all high-priority (red) transmissions to the hospital within 24 hours of transmission review, and 96.4% of the amber transmissions were reported within 48 hours. Overall, when we consider the additional time from transmission reception to transmission review and the time from review to communication, 91.7% of red transmissions were reported within 1 working day and 95.4% of amber transmissions within 2 working days (Figure 3).

#### Prioritized Events Management

Red transmissions required urgent visit or hospitalization in 60% (9/15) of the cases, whereas 92% (81/88) of amber events were managed totally remotely (Table 3). Most of the remotely managed events were related to an already treated arrhythmia or lung fluid impedance-related events.

**Table 3:** Clinical response to reported events.

Clinical response	Total (N=103), n (%)	Amber (n=88), n (%)	Red (n=15), n (%)
Heath care utilization required	16 (15.5)	7 (8.0)	9 (60.0)
Hospitalization for device replacement	7 (6.8)	4 (4.5)	3 (20.0)
Hospitalization for lead revision	2 (1.9)	—^a^	2 (13.3)
Hospitalization for cardiovascular reasons	1 (1.0)	—	1 (6.7)
In-office visit required	6 (5.8)	3 (3.4)	3 (20.0)
Event resolved remotely	87 (84.5)	81 (92.0)	6 (40.0)
New transmission required	17 (16.5)	11 (12.5)	6 (40.0)
Not urgent action^b^	70 (68.0)	70 (79.5)	—

^a^Not applicable.

^b^Event previously managed, monitoring the status of the event. The proportions are calculated on the total number of reported events (103, 88 amber, and 15 red).

### Efficiency: Comparison With the Benchmark Phase

**Figure 4:**
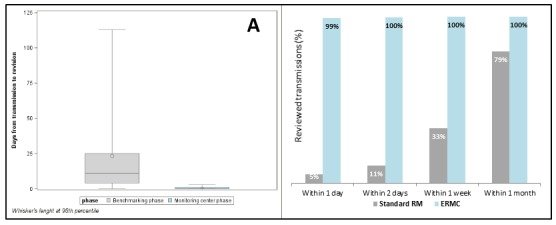
(A) Distribution of time from transmission to review, benchmarking phase versus external remote monitoring center (ERMC) phase; and (B) Percentage of reviewed transmissions, benchmarking phase versus ERMC phase. RM: remote monitoring.

Among the 153 patients followed by ERMC, 126 were remotely managed in the hospital from June 2015 to December 2015. In the ERMC phase, the median time to review was significantly reduced from 11 days (Q1-Q3: 4-25 days) to less than 24 hours (Q1-Q3: 0-1 day; Figure 4). During the standard follow-up phase, 21% of the transmissions had not been reviewed after 1 month, whereas during the monitoring center phase, all the transmissions were reviewed within 2 working days (Figure 4).

During the ERMC phase, patients were more compliant to the remote transmissions schedule than in the benchmarking phase, and the total number of annual transmissions per 100 patients increased from 350 to 608, respectively (*P*<.001). Nevertheless, only 78 (21.2%) transmissions required escalation to hospital staff, thus reducing the number of transmissions to review by 75% (IRR 0.25; 95% CI 0.66-0.81; *P*<.001). All data, separated by device type, are reported in Table 4.

**Table 4:** Rate of reviewed transmissions, benchmarking phase versus external remote monitoring center phase.

Device type	Benchmarking phase			Monitoring center phase			*P* value
	Total exposure time (years)	Reviewed TX^a^, n	Annual rate of hospital physician reviewed TX per 100 patient-years (95% CI)	Total exposure time (years)	Reviewed TX, n	Annual rate of hospital physician reviewed TX per 100 patient-years (95% CI)	
Overall (patients, n=126)	105	368	350 (316-387)	89	78^b^	88 (69-109)	<.001
CRT-D^c^ (n=55)	41	214	527 (461-603)	39	42	108 (78-146)	<.001
ICD^d^ (n=12)	10	35	366 (263-509)	8	6	75 (28-163)	<.001
IPG^e^ (n=58)	54	115	213 (177-255)	41	5	12 (4-28)	<.001
CRT-P^f^ (n=1)	1	4	411 (154-1096)	1	0	—^g^	—

^a^TX: transmissions.

^b^10 (14.7%) were classified as red.

^c^CRT-D: cardiac resynchronization therapy defibrillator.

^d^ICD: single- or dual-chamber implantable cardioverter defibrillator.

^e^IPG: single- or dual-chamber pacemaker.

^f^CRT-P: cardiac resynchronization therapy pacemaker.

^g^Not applicable.

## Discussion

### Principal Findings

This research showed that externalizing part of RM follow-up is safe, effective, and efficient in supporting a hospital previously challenged to guarantee high-quality standards of RM follow-up in terms of (1) time to review transmissions, both scheduled and unscheduled, to enable timely medical action as necessary; (2) dedicated staff and facility time to perform RM; and (3) patient compliance to RM, measured as the rate of transmissions per year.

### Safety and Practicability of External Remote Monitoring Center

The ERMC’s staff reviewed and managed all high-priority transmissions within 2 hours and 96.4% of the low-priority events within 2 working days, escalating only 15.7% of all transmissions to hospital staff due to a prioritized event being detected. The frequency of prioritized events was relatively high compared with the 8.2% presented by Cronin et al [14], but a direct comparison is challenging because of different approaches depending on the target population and, above all, because different variables, for example, learning curve, need to be taken into consideration when a third party is involved in the monitoring pathway. Once referred to the hospital staff, 15.5% of escalated transmissions led to a clinical action, in line with the 15.4% reported by Facchin et al [1], showing that ERMC is a precious tool to triage patients implanted with a CIED and to screen relevant events requiring clinical intervention. A total of 84.5% of escalated events did not lead to medical action but were nevertheless essential to hospital staff to monitor the evolution of patient clinical condition with respect to their ongoing treatment and medical history, in line with the definition of prioritized events in the protocol.

### Efficiency of External Remote Monitoring Center

It is well known that RM is a valuable tool that is able to support the follow-up of patients with an implanted CIED; however, it requires organizational changes in the hospital workflow to achieve optimal patient follow-up [16]. As such, reducing the time to review remote transmissions to a minimum is an essential aspect to achieve the full benefits of RM for optimal quality of care by enabling fast medical action [20]. Although staff reorganization is essential when aiming for optimal in-hospital RM management, there was no specific RM protocol in the hospital to follow up patients in a systematic way. To avoid bias and to expose all the possible challenges [20] of RM management, an RM protocol was not introduced during the benchmarking phase. In the 6-month period preceding the externalization of RM management, only one-third of the transmissions were processed within 1 week. During the ERMC phase, all transmissions were reviewed within 2 working days, and when a prioritized event occurred, the salient information was promptly communicated to the hospital. This result is comparable with the 2 to 4 days from actionable event onset to related clinical decision required in a number of previous controlled experiences [3,5].

Ensuring patient compliance is another key component to optimal follow-up, especially as patients can get disconnected from the system and may need support to reconnect. Moreover, with time, patient attention can decrease during follow-up and the use of RM may become intermittent. Our data showed that during the ERMC phase, the total number of remote transmissions increased by 74% as the monitoring center also communicated with the technical team to ensure that all patients would remain connected. This can also contribute to avoid variability in care between patients as well as encourage patient engagement in follow-up.

Regarding staff burden, with the escalation of prioritized events only, even with the large increase in the number of transmissions, the proportion of transmissions requiring review was reduced by 75%. If we apply the time to review transmissions (including administrative time) considered in Cronin et al [14], 21 min for prioritized events and 10 min for other transmissions, and the repartition of prioritized or other events found in the ERMC phase (21.2%/78.8%) to the benchmark phase, staff time required to follow up 100 patients would be reduced from 72 to 27 hours per year (62% reduction). Staff burden reduction can have an important organizational impact for the hospital as highly skilled health care professionals may then devote more time to treat more patients in need, thus optimizing patient access to care. It can also contribute to facilitate the implementation of best practice recommendations for follow-up of patients [16] as RM often requires important reorganization that hospitals with limited resources cannot achieve, in the sense of a more extensive and differentiated role organization. The presented research experience has been conducted in a small Italian hospital where cardiologists have to deal with the screening of all remote transmissions. In such a case, the cost of RM triage externalizations would represent an efficient, that is, a cost-saving, alternative and would save cardiologists’ time for more important clinical tasks [21].

### Limitations

We reported results of a single center experience, sharing the problems with efficacy and efficiency of the RM in a hospital where there was no standard center-specific protocol established for RM apart from the Heart Rhythm Society recommendations. Moreover, our practice may not be the standard of care across different health care systems. Further studies are required to deeply investigate if an ERMC strategy will be recommended in centers with a larger number of monitored patients and with predefined RM strategies.

Due to the limited sample size, we were not able to identify specific subgroups more eligible than others to receive external remote monitoring triage. Future studies could possibly be designated to address the topic.

Once the ERMC phase was completed, we noted that some areas of improvement are still required in the process of externalization of RM, such as refining prioritization of events based on ongoing medical therapy (eg, oral anticoagulant therapy).

Whether the externalization of RM management is able to improve the adherence to guidelines and recommendations and its effects on clinical outcome were not in the scope of this study.

### Conclusions

This experience in Cefalù Hospital’s cardiology department demonstrated that outsourcing part of the remote follow-up of patients through an ERMC is safe, effective, and efficient compared with standard RM performed at a hospital level. All the transmissions were reviewed within 2 working days and prioritized events were communicated promptly by ERMC, leading to a faster review of important events by hospital staff without the triaging burden. In a scenario of limited resources, such externalization of RM could be a key tool to save dedicated staff and facility time for more crucial patient care activities.

## Abbreviations

CIED: cardiac implantable electronic device
CLN: CareLink network
CRT-D: cardiac resynchronization therapy defibrillator
CRT-P: cardiac resynchronization therapy pacemaker
ERMC: external remote monitoring center
ICD: implantable cardioverter defibrillator
IPG: single- or dual-chamber pacemaker
IRR: incidence rate ratio
RM: remote monitoring
